# A Long-Term Performance Enhancement Method for FOG-Based Measurement While Drilling

**DOI:** 10.3390/s16081186

**Published:** 2016-07-28

**Authors:** Chunxi Zhang, Tie Lin

**Affiliations:** School of Instrumentation Science and Opto-Electronics Engineering, Beihang University, Beijing 100191, China; zhangchunxi@buaa.edu.cn

**Keywords:** measurement while drilling (MWD), fiber optic gyroscope (FOG), inertial navigation system (INS), minimum curvature method (MCM), Kalman filter

## Abstract

In the oil industry, the measurement-while-drilling (MWD) systems are usually used to provide the real-time position and orientation of the bottom hole assembly (BHA) during drilling. However, the present MWD systems based on magnetic surveying technology can barely ensure good performance because of magnetic interference phenomena. In this paper, a MWD surveying system based on a fiber optic gyroscope (FOG) was developed to replace the magnetic surveying system. To accommodate the size of the downhole drilling conditions, a new design method is adopted. In order to realize long-term and high position precision and orientation surveying, an integrated surveying algorithm is proposed based on inertial navigation system (INS) and drilling features. In addition, the FOG-based MWD error model is built and the drilling features are analyzed. The state-space system model and the observation updates model of the Kalman filter are built. To validate the availability and utility of the algorithm, the semi-physical simulation is conducted under laboratory conditions. The results comparison with the traditional algorithms show that the errors were suppressed and the measurement precision of the proposed algorithm is better than the traditional ones. In addition, the proposed method uses a lot less time than the zero velocity update (ZUPT) method.

## 1. Introduction

In the oil industry, borehole trajectories needs to be measured accurately in drilling engineering and geological work [1,2]. Measurement-while-drilling (MWD) surveying systems provide the position and the orientation of the bottom hole assembly (BHA) in real-time during drilling. Present MWD surveying systems consist of three-axis accelerometers and three-axis magnetometers mounted inside special non-magnetic drill collars [3]. However, using magnetometers has a deleterious effect on the overall accuracy of the surveying process, since the drill string components contain magnetic interference, geomagnetic influences, and downhole ore deposits, which are randomly located and cannot be predicted [1,2]. This magnetic interference effect is reduced, but not eliminated, by utilizing long lengths of non-magnetic drill collars, but this solution increases the drilling technology costs due to the relatively high cost of these non-magnetic materials [4]. Meantime, the MWD surveying system is not capable of monitoring the drill bit in time since the MWD needs to be installed at least 15 m behind the drill bit due to the non-magnetic drill collar use [1].

As the inertial navigation system (INS) is free from magnetic interference effects, it has been proposed as an alternative to magnetometer-based MWD surveying [5,6,7]. Many scholars have done a lot of research work about gyroscope-based MWD. Reference [8] proposed a novel non-linear error model (NNEM) to reduce the propagated errors under large-angle attitude error conditions. Meantime, the particle filter (PF) and Kalman filter (KF) were compared. In [9] an in-drilling alignment (IDA) method was proposed to improve the surveying accuracy. References [10,11,12] studied the error models for gyroscope-based MWD. An alternative method is micro-electro-mechanical Systems (MEMS) gyroscope-based INS [1,8,9]. The advantage of MEMS gyroscopes is that they are very small, and it can easily meet the size requirements. However, MEMS gyroscopes have limited practical application due to their low precision compared with other gyroscopes. Fiber optic gyroscopes have many advantages over MEMS gyroscopes, such as shock and vibration force resistance, immunity from magnetic interference, and high reliability. These advantages make FOG-based inertial measurement units (IMUs) perfect candidates for MWD surveying systems, and this has attracted much interest [6,7,13]. Two limiting factors should be taken into account when applying FOG-based IMU in MWD: (1) the instrument size limitation and (2) the INS unlimited error growth. To accommodate the size, a single FOG system and dual FOG system combined with three orthogonal accelerometers has been proposed [5,13]. The single FOG system needs to stop drilling to keep static for surveying, thus continuous surveying cannot be realized. Dual-axis FOG system provides continuous surveying for the near vertical and the radical section of the well [4], while it cannot realize the entire attitude while surveying. Using a completely FOG-based IMU is a suggested alternative to the dual-FOG approach. On the other hand, it is clearly known that the position, velocity, and attitude errors continuously grow if there is no external observation to update the INS. For long-term and high accuracy surveying of FOG-based MWD, the zero velocity update (ZUPT) method is adopted [14]. Nevertheless, previous research demonstrated that ZUPT is time consuming [15]. As an alternative to ZUPT, the in-drilling alignment (IDA) method has been previously proposed and theoretically demonstrated as an approach for limiting the error growth [9,16]. However, the IDA method cannot be used easily, because of both the IDA method and the downhole drilling condition complexities.

From the above analysis, the main motivation of this paper is to develop a FOG-based MWD surveying system for well logging by using tri-axial FOG and tri-axial accelerometer. Furthermore, to suppress the INS error growth, a long-term surveying method is proposed based on the drilling feature. Finally, the semi-physics simulation is conducted to verify the proposed method based on the FOG-based MWD prototype.

## 2. Theory of FOG-Based Measurement While Drilling

### 2.1. Overall Design of FOG-Based MWD

**Figure 1:**
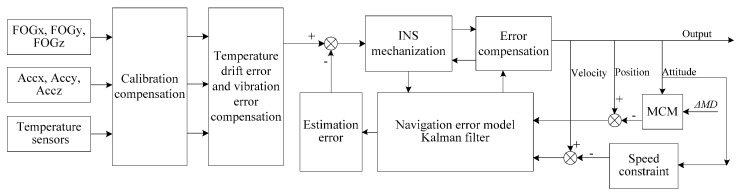
Schematic of the surveying method.

Figure 1 is the overall design of FOG-Based MWD. It contains three FOGs, three accelerometers and six temperature sensors. First of all, the inertial sensors collect the motion information of the MWD. Then, the sensor data are compensated for the removal of the fixed bias, temperature drift error, vibration error, etc. The bias and scale factor are obtained according to the model and calibration method which are described in the literature [17]. According to the characteristics of MWD, the temperature data is obtained by the slow self-heating, and the temperature drift error model is established using the multiple linear regression method. The vibration error model is established using grey neural network theory. Finally, the velocity, position and attitude of the MWD are obtained by inertial navigation algorithm. In order to suppress the error growth, the Kalman filter (KF) is used for integrated surveying algorithm. The estimation errors include random bias of FOGs, random bias of accelerometers, velocity error, position error and attitude error. All the estimation errors are compensated in real time. Section 2.4.2 corresponds to INS Mechanization block in the schematic of Figure 1. In this paper, the focus is the error growth with time, so the process and the methods of temperature and vibration error compensation are not discussed in detail.

The novelty of the proposed method is that two features of drilling are adopted as external observations. One is the drilling pipe length, and the other is the speed constraint while drilling. The proposed method will be described in detail in Section 3.

### 2.2. Structure of The Developed FOG-Based MWD

The MWD prototype developed in this paper is based on a FOG IMU. It is composed of three FOGs and three flexible quartz accelerometers arranged in three mutually orthogonal directions. Three-axis FOG is used to provide the 3D angular velocity measurements of the body, while the three-axis accelerometer is used to provide the 3D acceleration measurements of the body.

**Figure 2:**
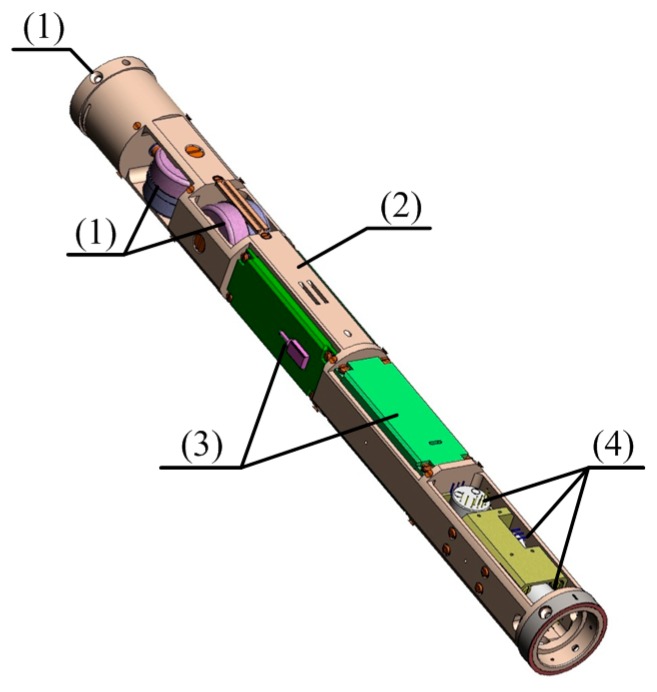
3D graphic model of the complete FOG-based IMU.

In the oil industry, the size of the MWD surveying system is very restricted, and three full FOGs cannot be directly installed. In this work, the three-axis integrated and flexible manufacturing technology is developed to design the IMU. The three-axis FOG has only one light source, which reduces the component size to satisfy the size requirement and also reduces power consumption. Moreover, the fiber rings, processing circuits, and light source are arranged independently along the mechanical body. Consequently, the FOGs have better temperature performance. This novel design method improves the FOGs performance. The IMU integrative structure is shown in Figure 2, where (1) is the module of 3-D fiber ring; (2) is the mechanical body; (3) is the module of processing circuit of 3-D FOG; (4) is the module of 3-D accelerometer. One light source was installed under the processing circuit.

### 2.3. Hardware Design

The hardware of the FOG-based MWD is composed of three FOGs, three accelerometers, accelerometer signal acquisition circuit, and navigation computer. The FOG is specially designed according to MWD requirement by our laboratory, and the model of the accelerometers is QZ-25A (Tianxinfangzhou Electronic Technology Co. Ltd., Beijing, China). The main performance parameters of the accelerometers are shown in Table 1.

**Figure 3:**
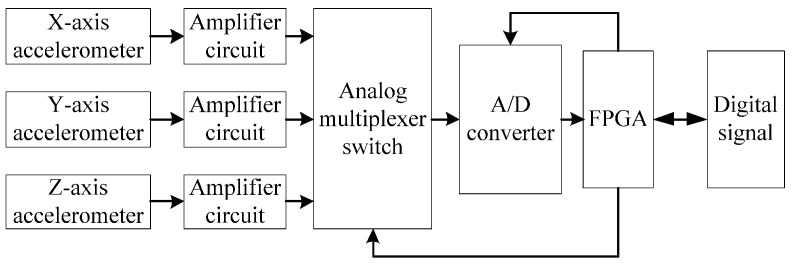
Principle of the accelerometer signal acquisition.

The principle of the accelerometer signal acquisition is shown in Figure 3. First of all, the output of accelerometer is sampled and amplified. Secondly, one of the three signals is chosen by the analog multiplexer switch and converted by the A/D converter. Thirdly, after the conversion is complete, the three digital signals are outputted by FPGA. The analog multiplexer switch and A/D converter are controlled by the FPGA.

**Figure 4:**
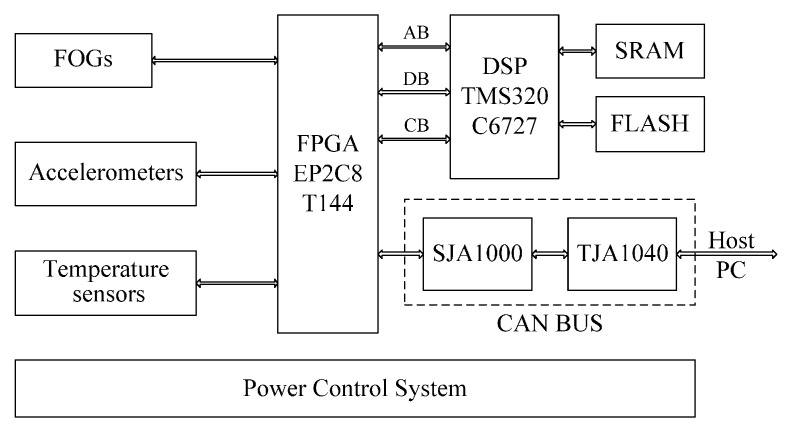
Block diagram of navigation computer.

The navigation computer is mainly utilized to collect all sensor data, preprocess data and run the navigation algorithm. The principle of the navigation computer is shown in Figure 4. The floating-point digital signal processor (DSP) is chosen as the navigation computer processor. The DSP has a high processing speed and can run complex navigation algorithms. A high performance core Field Programmable Gate Array (FPGA) is chosen as data acquisition and input/output (I/O) interfaces for output data of gyroscopes, accelerometers and temperature sensors. The CAN bus is used as the interface of the MWD surveying system.

### 2.4. Mathematical Calculation

#### 2.4.1. Relationship between MWD Body Coordinates and Navigation Coordinates

**Figure 5:**
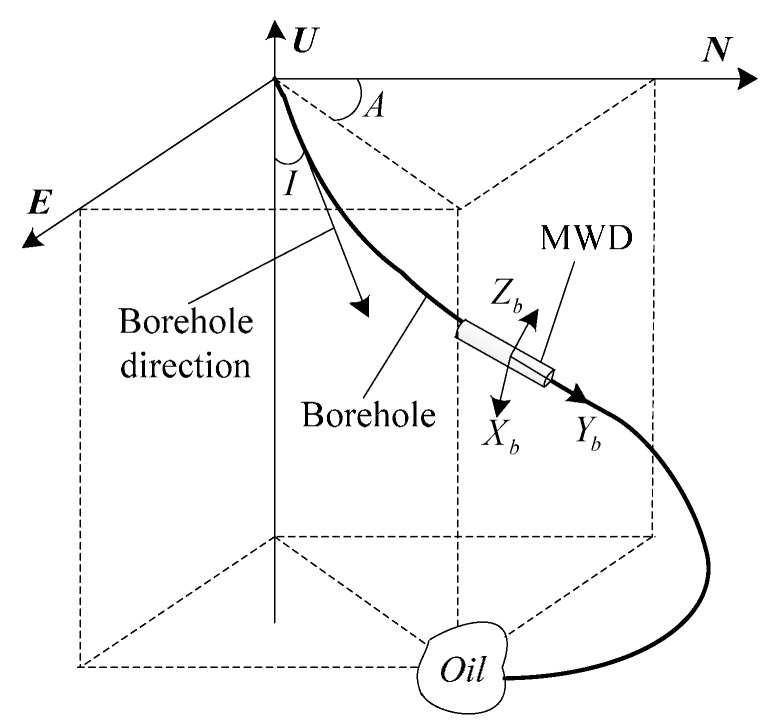
Coordinate and attitude angle diagram.

As shown in Figure 5, the *ENU* navigation coordinates are defined as east-north-up based on the right-hand rule and the *X_b_Y_b_Z_b_* MWD body coordinates are defined as right-forward-up based on the right-hand rule. The inclination angle (*I*) is the angle between the *Y_b_* axis and the vertical direction, and the azimuth angle (*A*) is the angle between the horizontal projection of *Y_b_* axis and the north.

**Figure 6:**
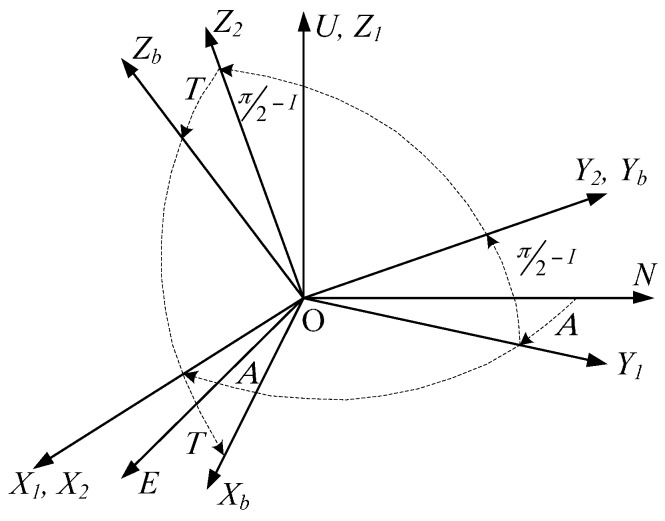
The coordinate transformation process.

The device transforms a fixed location to the current location through a rotation matrix. As shown in Figure 6, the navigation coordinates are used as a reference frame with a positive clockwise rotation. First, an angle of *A* rotates around $\vec{OU}$ to the coordinates *X*_1_*Y*_1_*Z*_1_, then an angle of π/2−I rotates around $\vec{OX_{1}}$ to the coordinates *X*_2_*Y*_2_*Z*_2_, and an angle of *T* rotates around $\vec{OY_{2}}$ to the coordinates *X_b_Y_b_Z_b_* which are the device body coordinates. Here *T* is the toolface angle that indicates the MWD instrument rotation around the $\vec{OY_{b}}$ axis.

Therefore, the rotation matrix is expressed as Equation (1), and the relationship between MWD body and navigation coordinates is expressed by Equation (2) [17]:

(1) $$C_{n}^{b}=R_{T}R_{I}R_{A}=[\begin{array}{ccc} \cos T\cos A+\sin T\sin A\cos I & -\cos T\sin A+\sin T\cos A\cos I & -\sin T\sin I\\ \sin A\sin I & \cos A\sin I & \cos I\\ \sin T\cos A-\cos T\sin A\cos I & -\sin T\sin A-\cos T\cos A\cos I & \cos T\sin I \end{array}$$

(2) $$\left[\begin{array}{c} x_{b}\\ y_{b}\\ z_{b} \end{array}\right]=C_{n}^{b}\left[\begin{array}{c} x_{n}\\ y_{n}\\ z_{n} \end{array}\right]$$

where the subscript *n* denotes the navigation frame, while superscript *b* denotes the body frame.

The inclination angle *I*, azimuth angle *A* and toolface angle *T* can be obtained by Equation (3):

(3) $$\{\begin{array}{l} I=\arcsin\sqrt{C_{1,3}\cdot C_{1,3}+C_{3,3}\cdot C_{3,3}}\\ A=\arctan\left(C_{2,1}/C_{2,2}\right)\\ T=-\arctan\left(C_{1,3}/C_{3,3}\right) \end{array}$$

where $C_{i,j}$ represents the row *i*, column *j* element of the matrix $C_{n}^{b}$.

#### 2.4.2. Inertial Navigation Algorithm

The magnetometer-based MWD surveying system only provides the azimuth and the inclination of the BHA, and the position is determined using the drill pipe length. In contrast, the FOG-based MWD provides both the attitude and the position.

The classical inertial navigation algorithm is described as [18]:

(4) $$\{\begin{array}{l} {\dot{C}}_{b}^{n}=C_{b}^{n}\left(\left(\omega_{ib}^{b}-C_{n}^{b}\left(\omega_{ie}^{n}+\omega_{en}^{n}\right)\right)\times \right)\\ {\dot{v}}^{n}=C_{b}^{n}f^{b}-\left(2\omega_{ie}^{n}+\omega_{en}^{n}\right)\times v^{n}+g^{n}\\ \dot{L}=v_{N}^{n}/\left(R+h\right)\\ \dot{\lambda }=v_{E}^{n}\sec L/\left(R+h\right)\\ \dot{h}=v_{U}^{n} \end{array}$$

where the body angular rate $\omega_{ib}^{b}={\left[\begin{array}{ccc} \omega_{x}^{b} & \omega_{y}^{b} & \omega_{z}^{b} \end{array}\right]}^{T}$ is measured by FOGs. The Earth rotation rate vector $\omega_{ie}^{n}={\left[\begin{array}{ccc} 0 & \omega_{ie}\cos L & \omega_{ie}\sin L \end{array}\right]}^{T}$ is in the navigation frame. $\omega_{en}^{n}={\left[\begin{array}{ccc} -v_{N}^{n} & v_{E}^{n} & v_{E}^{n}\tan L \end{array}\right]}^{T}/(R+h)$ is the angular rate of the navigation frame with respect to the Earth frame, expressed in the navigation frame. $v^{n}={\left[\begin{array}{ccc} v_{E}^{n} & v_{N}^{n} & v_{U}^{n} \end{array}\right]}^{T}$ is the ground velocity in the navigation frame coordinates, which the subscripts *E*, *N*, and *U* stand for east, north and upward velocity components, respectively. $f^{b}={\left[\begin{array}{ccc} f_{x}^{b} & f_{y}^{b} & f_{z}^{b} \end{array}\right]}^{T}$ is the accelerometers’ output specific force. $g^{n}={\left[\begin{array}{ccc} 0 & 0 & -g \end{array}\right]}^{T}$ is the gravity vector in the navigation frame and *R* is the radius of the Earth. The positions *L*, λ, and *h* of MWD are the latitude, longitude, and height, respectively. The 3×3 matrix (×) represents the vector cross product. For example, when $a={\left[\begin{array}{ccc} a_{1} & a_{2} & a_{3} \end{array}\right]}^{T}$ and $b={\left[\begin{array}{ccc} b_{1} & b_{2} & b_{3} \end{array}\right]}^{T}$, then $a\times b=\left[\begin{array}{c} a_{2}b_{3}-a_{3}b_{2}\\ a_{3}b_{1}-a_{1}b_{3}\\ a_{1}b_{2}-a_{2}b_{1} \end{array}\right]=\left[\begin{array}{ccc} 0 & -a_{3} & a_{2}\\ a_{3} & 0 & -a_{1}\\ -a_{2} & a_{1} & 0 \end{array}\right]\left[\begin{array}{c} b_{1}\\ b_{2}\\ b_{3} \end{array}\right]=(a\times )b$, so, $(a\times )=\left[\begin{array}{ccc} 0 & -a_{3} & a_{2}\\ a_{3} & 0 & -a_{1}\\ -a_{2} & a_{1} & 0 \end{array}\right]$.

## 3. The Long-Term Surveying Method

The INS-based MWD has many advantages over magnetometer-based MWDs, but exhibits an unlimited growth of the position, velocity, and attitude errors if there is no external observation to update the surveying system. For long-term and high accuracy surveying, there are other kinds of systems such as global positioning system (GPS), odometer, and celestial navigation that are integrated with INS [19,20,21] to suppress the growing errors of INS on the ground or in space. However, the working underground condition limits the integration with the navigation systems mentioned above, so the available information to enhance the INS performance should be found.

In this section, we will build the error model, and then find the available external information through the drilling characteristic analysis. Finally, we realize the proposed algorithm through the Kalman filter design.

### 3.1. FOG-Based MWD Error Model

The relationship between the true value and computed value of attitude, velocity and position of INS is given as the following expressions [17]:

(5) $$\{\begin{array}{l} {\bar{v}}^{n}=v^{n}+\delta v^{n}\\ {\bar{C}}_{b}^{n}=\left[I-\left(\varphi \times \right)\right]C_{b}^{n}\\ {\bar{C}}_{e}^{n}=\left[I-\left(\delta \theta \times \right)\right]C_{e}^{n}\\ \bar{h}=h+\delta h \end{array}$$

where ${\bar{v}}^{n}$ is the computed velocity, $\delta v^{n}$ is the velocity error. ${\bar{C}}_{b}^{n}$ is the computed body to navigation frame transformation matrix, and $C_{b}^{n}$ is the true matrix. φ is the attitude error (δI, δT and δA), and I is the identity matrix. ${\bar{C}}_{e}^{n}$ is the Earth-fixed computed direction cosine matrix to navigation frame transformation, $C_{e}^{n}$ is the true matrix, and δθ is the position error (δL and δλ). Lastly, $\bar{h}$ is computed altitude, h is the true altitude, and δh is the altitude error.

According to Equations (4) and (5), the FOG-based MWD error model is represented as [17]:

(6) $$\left\{ \begin{array}{l} \delta {\dot{v}}^{n}=-\left(\delta \omega_{en}^{n}+2\delta \omega_{ie}^{n}\right)\times v^{n}-\left(\omega_{en}^{n}+2\omega_{ie}^{n}\right)\times \delta v^{n}+f^{n}\times \varphi +C_{b}^{n}\delta f^{b}+\delta g^{n}\\ \dot{\varphi }=\delta \omega_{en}^{n}+\omega_{ie}^{n}\times \delta \theta +\varphi \times \left(\omega_{en}^{n}+\omega_{ie}^{n}\right)-\varepsilon^{n}\\ \delta \dot{\theta }=\delta \omega_{en}^{n}-\omega_{en}^{n}\times \delta \theta \\ \delta \dot{h}=\delta v_{U}^{n} \end{array}\right.$$

where $\delta \omega_{ie}^{n}$ is the Earth rotation rate error and $\delta g^{n}$ is the gravity vector error. $\delta f^{n}={\left[\begin{array}{ccc} \delta f_{E} & \delta f_{N} & \delta f_{U} \end{array}\right]}^{T}$ is the accelerometer error in the navigation frame and $\varepsilon^{n}={\left[\begin{array}{ccc} \varepsilon_{E} & \varepsilon_{N} & \varepsilon_{U} \end{array}\right]}^{T}$ is the gyroscope error in the navigation frame.

### 3.2. Method of Integrated Navigation

During drilling, the MWD instrument is installed with the BHA and moves with the drill pipe. There are two features for the drilling process. One is the instrument velocity, and only the velocity in the axial direction of the instrument (y-axis) is not zero due to space limitation; the velocity x-axis and z-axis can be approximately regarded as zero. Therefore, the constraints under ideal conditions are as follows:

(7) $$\{\begin{array}{c} v_{bx}^{b}=0\\ v_{bz}^{b}=0 \end{array}$$

The other is the connecting pipe length [1]. The position of the BHA is determined by the attitude angles assuming a certain trajectory between the surveying stations. The common calculation methods [22,23] of well trajectory are shown in the Table 2. The average angle method (AAM) assumes that the measuring section is a straight line, and the direction of the well is a vector of the two measuring points. The balance tangent method (BTM) assumes that the measuring section is a line which is composed of half of the length of the two measuring section, and the direction of the well is consistent with the direction of the upper and lower measuring points. The corrected average angle method (CAAM) assumes that the measuring section is a cylindrical spiral, and spiral points at both ends are tangent to the upper and lower. The minimum curvature method (MCM) assumes that the measuring section is a circular arc on the plane, and at both ends of the circular are tangent to the upper and lower borehole direction. The chord step method (CSM) assumes that the measuring section is a circular arc on the plane, and the length of measuring section is as chord length.

**Figure 7:**
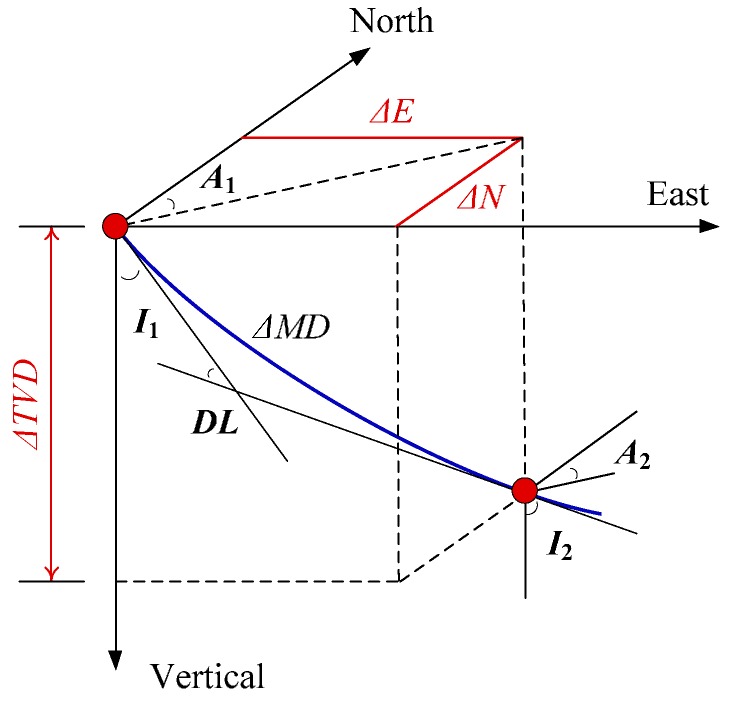
MCM stationary survey.

Reference [22] analyzed the calculation errors of these methods and noted that it exhibits a certain similarity between different methods of calculation of error. In [23] it was pointed out that the precision of curve methods is higher than that of the straight line and broken line method, as the assumption of the curve method is more reasonable in practical applications. Therefore, the CAAM, MCM and CSM have the highest accuracy, and the error between them is very small. Meantime, as [23] points out “the minimum curvature method and the chord step method are suitable for the well section of the underground power drill. Corrected average angle method is suitable for rotary drilling sections”. Therefore, we choose the MCM to calculate the trajectory. MCM [24,25] assumes the two surveying stations lie on a circular arc, and the arc is located in a plane for which the orientation is known at both ends by knowing the inclination and azimuth angles. Figure 7 illustrates the MCM.

In Figure 7, inclination and azimuth angles at station 1 are denoted as *I*_1_ and *A*_1_, respectively, while the inclination and azimuth angles at station 2 are denoted as *I*_2_ and *A*_2_, respectively. The MCM fits a ΔMD spherical arc between the two stations by calculating the curvature “*DL*” from the 3D vectors and scaling by a ratio factor (*RF*). When the first station positions are known, the second station positions is computed using the following expressions [25]:

(8) $$\left\{ \begin{array}{l} DL=\arccos\left(\cos\left(I_{2}-I_{1}\right)-\sin I_{1}\sin I_{2}\left(1-\cos\left(A_{2}-A_{1}\right)\right)\right)\\ RF=2\tan\left(DL/2\right)/DL\\ \Delta TVD=1/2\Delta MD\left(\cos I_{1}+\cos I_{2}\right)RF\\ \Delta N=1/2\Delta MD\left(\sin I_{1}\cos A_{1}+\sin I_{2}\cos A_{2}\right)RF\\ \Delta E=1/2\Delta MD\left(\sin I_{1}\sin A_{1}+\sin I_{2}\sin A_{2}\right)RF \end{array}\right.$$

where ΔTVD  is the difference in the true vertical depth between the two stations with ΔN  and ΔE being the difference in the north and east directions, respectively. Δ*MD* is the drilling pipe length. Scale to 10 pt size and align correctly.

Therefore, those two features are adopted as external observations to aid the INS based on Kalman filter in this paper.

### 3.3. Kalman Filter Design

In this work, the Kalman filter is designed to conduct information fusion of the FOG-based INS algorithm results and external observations. Moreover, the Kalman filter estimated results are used to compensate the error of the FOG IMU and navigation output.

#### 3.3.1. State-Space System Model

The state-space system model is established from the navigation errors differential equations represented as Equation (9). Both the gyroscope and accelerometer errors are considered as the composition of bias error and white noise. The general linear stochastic system model is given by [26]:

(9) $$\left\{ \begin{array}{l} \dot{X}=FX+GW,\;W\sim N(0,Q)\\ X=[\delta L\;\delta \lambda \;\delta h\;\delta v_{E}\;\delta v_{N}\;\delta v_{U}\;\delta I\;\delta T\;\delta A\;\\ \;aB_{x}\;aB_{y}\;aB_{z}\;gB_{x}\;gB_{y}\;gB_{z}{]}^{T}\\ W={\left[0_{1\times 3}\;w_{a}^{T}\;w_{g}^{T}\;0_{1\times 6}\right]}^{T} \end{array}\right.$$

where X is the error states vector composed of navigation errors and inertial sensor bias errors, F is the dynamic matrix, G is the noise coefficient matrix, and W is the system noise vector consisting of the white noises of inertial sensors. We assumed that W has the normal distribution with the variance matrix Q. δL, δλ, and δh are the latitude error, longitude error, and height error, respectively; $\delta v_{E}$, $\delta v_{N}$, and $\delta v_{U}$ are the velocity errors in the east, north, and vertical directions, respectively; δI, δT, and δA are the errors of inclination angle, toolface angle and azimuth angle, respectively; $aB_{x}$, $aB_{y}$, and $aB_{z}$ are the accelerometer bias errors, respectively; $gB_{x}$, $gB_{y}$, and $gB_{z}$ are the gyroscope bias errors, respectively; $w_{a}$ is the accelerometer white noise matrix, and $w_{g}$ is the gyroscope white noise matrix. $0_{i\times j}$ represents an i×j zero matrix.

The detailed matrix of F and G is given in Equations (10)–(13), respectively. Ω is the rotational angular velocity of the Earth. $R_{M}$ and $R_{N}$ are the main curvature radiuses along the meridian, respectively:

(10) $$F=\left[\begin{array}{cc} {F^{1}}_{9\times 9} & {F^{2}}_{9\times 6}\\ 0_{6\times 9} & 0_{6\times 6} \end{array}\right]$$

(11) $${F^{2}}_{9\times 6}=\left[\begin{array}{ll} 0_{3\times 3} & 0_{3\times 3}\\ C_{b}^{n} & 0_{3\times 3}\\ 0_{3\times 3} & C_{b}^{n} \end{array}\right]$$

(12) $$G_{15\times 15}=\left[\begin{array}{llll} 0_{3\times 3} & 0_{3\times 3} & 0_{3\times 3} & 0_{3\times 6}\\ 0_{3\times 3} & C_{b}^{n} & 0_{3\times 3} & 0_{3\times 6}\\ 0_{3\times 3} & 0_{3\times 3} & C_{b}^{n} & 0_{3\times 6}\\ 0_{6\times 3} & 0_{6\times 3} & 0_{6\times 3} & 0_{6\times 6} \end{array}\right]$$

(13) $$\begin{array}{c} F_{9\times 9}^{1}=[\begin{array}{ccccc} 0 & 0 & \frac{-v_{N}}{{\left(R_{M}+h\right)}^{2}} & 0 & \frac{1}{R_{M}+h}\\ \frac{v_{E}\tan L\sec L}{R_{N}+h} & 0 & \frac{-v_{E}\sec L}{{\left(R_{N}+h\right)}^{2}} & \frac{\sec L}{R_{N}+h} & 0\\ 0 & 0 & 0 & 0 & 0\\ 2\omega_{ie}\left(v_{U}\sin L+v_{N}\cos L\right)+\frac{v_{E}v_{N}}{R_{N}+h}\sec^{2}L & 0 & \frac{v_{E}v_{U}-v_{E}v_{N}\tan L}{{\left(R_{N}+h\right)}^{2}} & \frac{v_{N}\tan L-v_{U}}{R_{N}+h} & 2\omega_{ie}\sin L+\frac{v_{E}}{R_{N}+h}\tan L\\ -2v_{E}\omega_{ie}\cos L-\frac{{v_{E}}^{2}}{R_{N}+h}\sec^{2}L & 0 & \frac{v_{N}v_{U}}{{\left(R_{M}+h\right)}^{2}}+\frac{{v_{E}}^{2}\tan L}{{\left(R_{N}+h\right)}^{2}} & -2\left(\omega_{ie}\sin L+\frac{v_{E}}{R_{N}+h}\tan L\right) & \frac{-v_{U}}{R_{M}+h}\\ -2v_{E}\omega_{ie}\sin L & 0 & -\frac{{v_{N}}^{2}}{{\left(R_{M}+h\right)}^{2}}-\frac{{v_{E}}^{2}}{{\left(R_{N}+h\right)}^{2}} & 2\left(\omega_{ie}\cos L+\frac{v_{E}}{R_{N}+h}\right) & \frac{2v_{N}}{R_{M}+h}\\ 0 & 0 & 0 & \frac{v_{N}}{{\left(R_{M}+h\right)}^{2}} & 0\\ -\omega_{ie}\sin L & 0 & \frac{-v_{E}}{{\left(R_{N}+h\right)}^{2}} & \frac{1}{R_{N}+h} & 0\\ \omega_{ie}\cos L+\frac{v_{E}}{R_{N}+h}\sec^{2}L & 0 & \frac{-v_{E}\tan L}{{\left(R_{N}+h\right)}^{2}} & \frac{\tan L}{R_{N}+h} & 0 \end{array}\\ \;\begin{array}{cccc} 0 & 0 & 0 & 0\\ 0 & 0 & 0 & 0\\ 1 & 0 & 0 & 0\\ -2\omega_{ie}\cos L-\frac{v_{E}}{R_{N}+h} & 0 & -f_{U} & f_{N}\\ -\frac{-v_{N}}{R_{M}+h} & f_{U} & 0 & -f_{E}\\ 0 & -f_{N} & f_{E} & 0\\ \frac{-1}{R_{M}+h} & 0 & \omega_{ie}\sin L+\frac{v_{E}}{R_{N}+h}\tan L & -\omega_{ie}\cos L-\frac{v_{E}}{R_{N}+h}\\ 0 & -\omega_{ie}\sin L-\frac{v_{E}}{R_{N}+h}\tan L & 0 & \frac{-v_{N}}{R_{M}+h}\\ 0 & \omega_{ie}\cos L+\frac{v_{E}}{R_{N}+h} & \frac{v_{N}}{R_{M}+h} & 0 \end{array}] \end{array}$$

#### 3.3.2. Observation Updates Model

The velocity of the MWD was selected as one of the external information. The x-axis and z-axis velocity was not zero because of the vibration interference. This interference is described as white noise:

(14) $$\left[\begin{array}{c} v_{bx,Virtual}^{b}\\ v_{bz,Virtual}^{b} \end{array}\right]=\left[\begin{array}{c} 0\\ 0 \end{array}\right]+\left[\begin{array}{c} \upsilon_{x}\\ \upsilon_{z} \end{array}\right]$$

where $\upsilon_{x}$ and $\upsilon_{z}$ are white noise.

The transformation of the velocity of the navigation frame to the body frame is described as:

(15) $$v_{b}=C_{n}^{b}v_{n}$$

Then, the velocity error is obtained by differentiating Equation (16):

(16) $$\begin{array}{l} \delta v^{b}=C_{n}^{b}\cdot \delta v^{n}+\delta C_{n}^{b}\cdot v^{n}\\ \;=C_{n}^{b}\cdot \delta v^{n}+E\cdot C_{n}^{b}v^{n}\\ \;=C_{n}^{b}\cdot \delta v^{n}+E\cdot v^{b} \end{array}$$

where ***E***, the attitude angle error antisymmetric matrix, is described as:

(17) $$E=\left[\begin{array}{ccc} 0 & -\delta A & \delta T\\ \delta A & 0 & -\delta I\\ -\delta T & \delta I & 0 \end{array}\right]$$

With Equations (1), (16) and (17), Equation (16) becomes:

(18) $$\left[\begin{array}{c} \delta v_{bx}^{b}\\ \delta v_{by}^{b}\\ \delta v_{bz}^{b} \end{array}\right]=\left[\begin{array}{ccc} \cos T\cos A+\sin T\sin A\cos I & -\cos T\sin A+\sin T\cos A\cos I & -\sin T\sin I\\ \sin A\sin I & \cos A\sin I & \cos I\\ \sin T\cos A-\cos T\sin A\cos I & -\sin T\sin A-\cos T\cos A\cos I & \cos T\sin I \end{array}\right]\left[\begin{array}{c} \delta v_{E}\\ \delta v_{N}\\ \delta v_{U} \end{array}\right]+\left[\begin{array}{ccc} 0 & v_{bz}^{b} & -v_{by}^{b}\\ v_{bz}^{b} & 0 & v_{bx}^{b}\\ v_{by}^{b} & -v_{bx}^{b} & 0 \end{array}\right]\left[\begin{array}{c} \delta I\\ \delta T\\ \delta A \end{array}\right]$$

where $v_{bx}^{b}$, $v_{by}^{b}$, and $v_{bz}^{b}$ is calculated using Equation (15).

Assuming the velocity calculated by the INS in the body frame is described as ${\left[\begin{array}{cc} v_{x,INS}^{b} & v_{z,INS}^{b} \end{array}\right]}^{T}$, Equation (19) can be obtained using Equation (18):

(19) [vx,INSbvz,INSb]=[00]+[δvbxbδvbzb][vx,INSbvz,INSb]=[cosTcosA+sinTsinAcosI−cosTsinA+sinTcosAcosI−sinTsinIsinTcosA−cosTsinAcosI−sinTsinA−cosTcosAcosIcosTsinI][δvEδvNδvU]+[00−vbybvbyb00][δIδTδA]

The differential between the velocity calculated by the INS in the body frame and the instrument velocity is:

(20) $$\left[\begin{array}{c} v_{x,INS}^{b}\\ v_{z,INS}^{b} \end{array}\right]-\left[\begin{array}{c} V_{bx,Virtual}^{b}\\ V_{bz,Virtual}^{b} \end{array}\right]=\left[\begin{array}{c} \delta v_{bx}^{b}\\ \delta v_{bz}^{b} \end{array}\right]-\left[\begin{array}{c} \upsilon_{x}\\ \upsilon_{z} \end{array}\right]$$

The differences ΔTVD, ΔN, and ΔE are obtained by MCM when drilling frequently stops. The stationary position ($L_{MCM}$, $\lambda_{MCM}$ and $h_{MCM}$) adopted as the other external information is calculated by Equation (21):

(21) $$\left\{ \begin{array}{l} L_{MCM}\left(k\right)=L_{0}+\sum_{i=1}^{k}\nabla N_{i}/\left(R_{M}\left(k\right)+h\left(k\right)\right)\\ \lambda_{MCM}\left(k\right)=\lambda_{0}+\sum_{i=1}^{k}\nabla E_{i}/\left(\left(R_{N}\left(k\right)+h\left(k\right)\right)\cdot \cos L_{MCM}\left(k\right)\right)\\ h_{MCM}\left(k\right)=h_{0}+\sum_{i=1}^{k}\nabla TVD_{i} \end{array}\right.$$

where $L_{0}$, $\lambda_{0}$, and $h_{0}$ are the initial latitude, longitude, and height, respectively.

Therefore, the measurement equation of the MWD motion-constraint-aided INS is described with Equations (19)–(21):

(22) $$Z_{k}=\left[\begin{array}{c} L_{INS}-L_{MCM}\\ \lambda_{INS}-\lambda_{MCM}\\ h_{INS}-h_{MCM}\\ \delta v_{bx}^{b}\\ \delta v_{bz}^{b} \end{array}\right]=\left[\begin{array}{c} \delta L\\ \delta \lambda \\ \delta h\\ \delta v_{bx}^{b}\\ \delta v_{bz}^{b} \end{array}\right]=H_{k}X+\upsilon$$

where υ is the measurement noise vector. $H_{k}$ is described as:

(23) $$H_{k}=\left[\begin{array}{cc} \begin{array}{ccc} 1 & 0 & 0\\ 0 & 1 & 0\\ 0 & 0 & 1 \end{array} & 0_{3\times 12}\\ 0_{2\times 3} & \begin{array}{llllll} \cos T\cos A+\sin T\sin A\cos I & -\cos T\sin A+\sin T\cos A\cos I & -\sin T\sin I & 0 & 0 & -v_{by}^{b}\\ \sin T\cos A-\cos T\sin A\cos I & -\sin T\sin A-\cos T\cos A\cos I & \cos T\sin I & v_{by}^{b} & 0 & 0 \end{array}\;0_{2\times 6} \end{array}\right]$$

## 4. Semi-Physics Simulation

**Figure 8:**
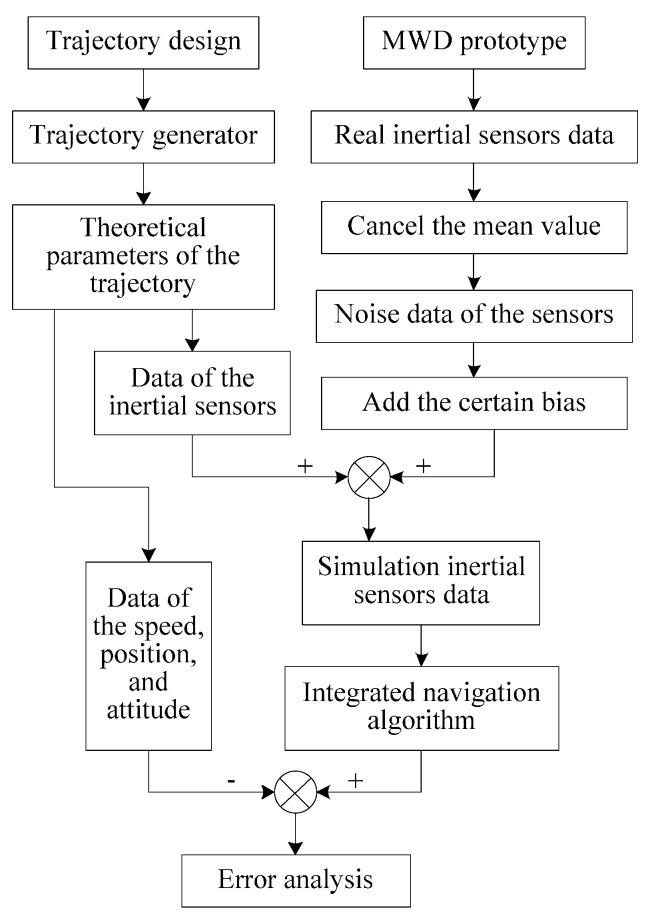
Flowchart of the experiment.

The initial evaluation of FOG-based MWD surveying system was conducted under laboratory conditions to validate the algorithm. Figure 8 shows the experimental process.

First of all, the trajectory of the oil borehole is designed, and the theoretical parameters of the trajectory are generated by a generator, including the three-axis angular velocity, three-axis acceleration, attitude, velocity, and position of the MWD. Secondly, the noise data of inertial sensors were acquired from the FOG-based MWD prototype. Thirdly, the simulation inertial sensor data were obtained from the theoretical three-axis angular velocity and acceleration added to the noise data, respectively. Then, using the simulation inertial sensors data, the errors produced by proposed method are compared with those produced by the traditional method.

### 4.1. Trajectory Design

**Figure 9:**
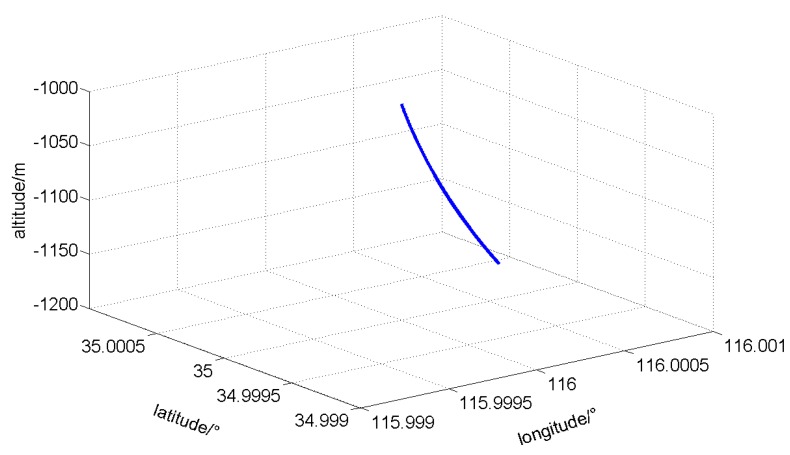
Three-dimensional figure of the oil borehole trajectory.

The parameters of the generated standard trajectory are as follows: the initial longitude is 116°, latitude is 35°, and altitude is −1000 m. The original azimuth angle is 180°, inclination angle is 20°, and the toolface angle is 0°. The time of the whole process is 5100 s, the move speed of MWD is 2 m/min along the drilling pipe, and every 5 min the MWD instrument stops 1 min (for ZUPT) [4,9]. The drilling pipe length is provided each 10 m. During the whole process, azimuth and toolface angles remain unchanged, while the inclination angle changed by 30°. The generated standard trajectory according to the conditions mentioned above is shown in Figure 9.

### 4.2. Get Noise Data of the Inertial Sensors

**Figure 10:**
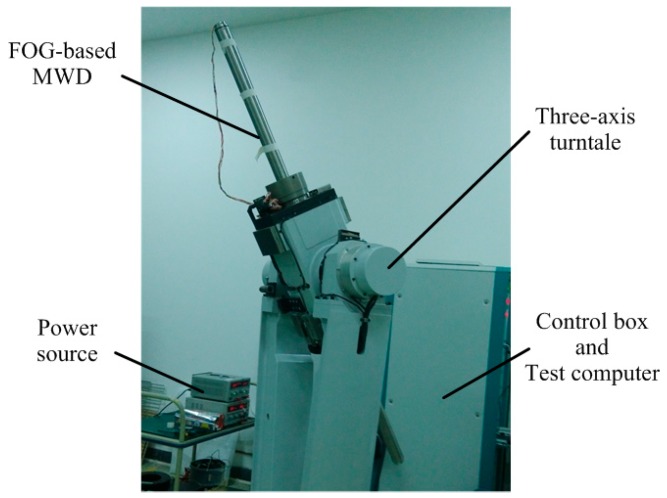
FOG-based MWD prototype testing.

The FOG-based MWD prototype is designed as in Section 2. The noise data of the inertial sensors were obtained from the prototype. The MWD prototype was installed on a three-axis turntable that was designed especially for the MWD instrument. The turntable provides accurate rotation around x-, y- and z-axis; meanwhile, the inclination, toolface, and azimuth angle of the MWD are changed, respectively. After the starting the MWD and turntable, we kept the MWD instrument at any attitude and collected the inertial sensor static data. The noise data was obtained by canceling the mean value from the collected data at a frequency of 100 Hz. Then, the bias (FOG: 0.2°/h, accelerometer: $1.0\times 10^{-3}$ m/s^2^) was added to the reserved noises of the gyroscopes and accelerometers, respectively. Figure 10 shows the testing process. Table 3 shows the designed sensor parameters of the FOG-based MWD prototype.

### 4.3. Experiment Results and Analysis

**Figure 11:**
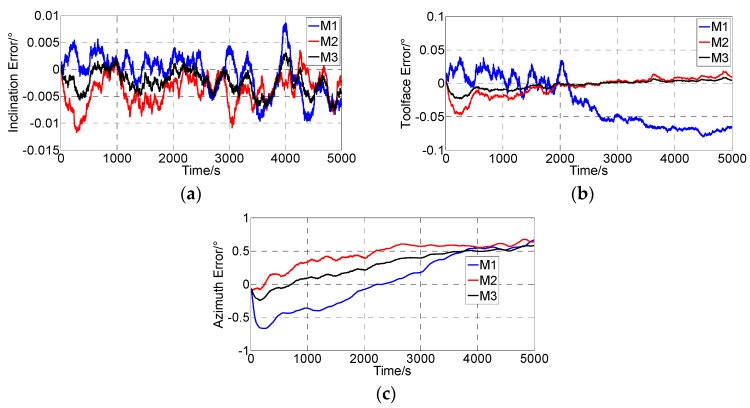
Attitude angle errors. (**a**) Inclination angle error; (**b**) Toolface angle error; (**c**) Azimuth angle error.

**Figure 12:**
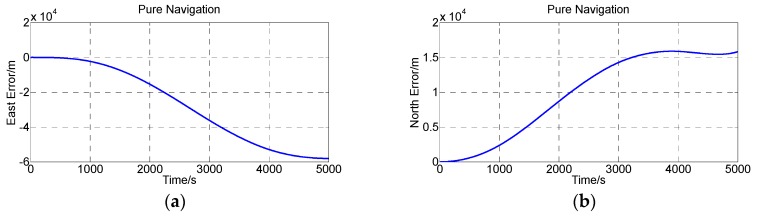
Position errors calculated by the pure navigation. (**a**) East error; (**b**) North error.

**Figure 13:**
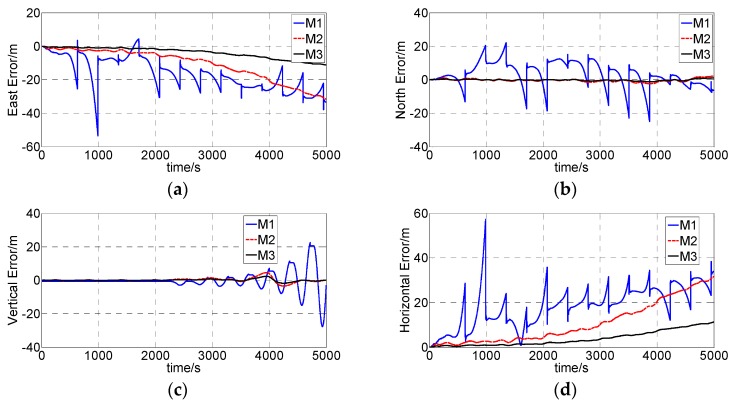
Position errors calculated by the integrated surveying algorithms. (**a**) East error; (**b**) North error; (**c**) Vertical error; (**d**) Horizontal error.

When the simulation data were obtained, the integrated surveying algorithm proposed in Section 3 was compared with the traditional algorithms (in Table 4) by simulation calculation. The method M2 is only using the drilling pipe length as the external information. The Figure 11 shows the attitude angle errors. The Figure 12 and Figure 13 show the position errors.

Both the inclination and the toolface errors were limited over time, while the azimuth error continued to increase. The main reason for such characteristics is that the external observation of all the methods is only concerned with acceleration. This drift in the azimuth angle appeared due to a FOG bias error, while the inclination and toolface angles are related to the accelerometer error more than the FOG error. The drift in the azimuth angle could not be compensated by the Kalman filter because the azimuth orientation is not coupled with the velocity or the position components. Conversely, the drifts in the inclination and toolface angles are compensated.

Only the velocity is adopted as an external observation for the ZUPT and the pipe length is adopted as external observation for the “Integrated with drilled pipe length”, while both the velocity and the pipe length were adopted as external observations for the proposed method. Thus, the attitude errors generated by the proposed method are smaller than the traditional methods. The maximum absolute attitude errors are 0.0077°, 0.0230° and 0.5832° , while the attitude errors generated by M1 are 0.0097°, 0.0802° and 0.6653°, the attitude errors generated by M2 are 0.0117°, 0.0470° and 0.6354° (Table 5).

The INS exhibits an unlimited growth error if there is no external observation to update the surveying system. As Figure 10 shows, during the simulation calculation, the east error achieves −58,106 m and the north error achieves 15,802 m. The proposed method and the comparison of the two methods can successfully suppress the error growth (Figure 12). No matter what method is adopted, the error cannot be eliminated clearly and the error will grow over time.

When using the ZUPT (M1), the velocity errors were limited near to zero, but the position errors drifted since the previous error in the velocities. After the ZUPT station, the velocity errors grew linearly with time due to not properly estimating the accelerometer bias errors. The position errors were obtained by integrating the corresponding velocity errors. The position error remained constant at the ZUPT station but grew with time between neighboring ZUPTs, and the errors exhibition growth grew during the whole process. Conversely, the errors calculated by M2 and M3 (proposed algorithm) were smooth and small compared with those calculated by ZUPT algorithm.

The error generated by the proposed algorithm is smaller than the M2 algorithm, especially the East error. The reason is that M2 adopted the pipe length as external observation only and the length was translated to velocity measurements update to the inertial sensors measurements, while the proposed method adopted both the velocity and the pipe length as external observations. When pipe length is translated to velocity, some noise is introduced, but the proposed method used the pipe length directly and calculated the position differences. Table 6, shows that the maximum absolute position errors (East, North, Vertical and Horizontal) generated by the proposed method were 11.23 m, 1.12 m, 2.34 m and 11.29 m, while M1’s position errors were 53.55 m, 22.05 m, 22.46 m and 57.32 m, and M2’s position errors were 31.60 m, 2.19 m, 4.56 m and 31.67 m.

The above analysis shows that the attitude measurement precision is at the same level, while the position measurement precision of the proposed algorithm is greater than the traditional algorithm. The ZUPT algorithm application effect is the worst of the three methods, which regularly needs to stop drilling and is time consuming. About 840 s of the whole simulation time (16.5%) is only for ZUPT. The other two algorithms do not need to interrupt the drilling process, and the proposed method has the highest precision of the three methods.

## 5. Conclusions

In the present study, an inertial navigation technique utilizing a commercially FOG-based IMU was proposed as a replacement for the presently used magnetometer-based surveying methods. It has wide application prospects for it is free from magnetic interference effects. In this study, a MWD instrument was manufactured by a new design method based on FOG and a quartz flexible accelerometer. An integrated surveying method was developed according to drilling features to suppress the errors and enhance the long-term performance. The results of the comparison with the traditional methods indicated that the proposed method in this paper successfully suppressed the error growth, especially has high positioning error. Thus, the proposed method improves the long-term performance of the FOG-based MWD. None of the algorithms can completely suppress the growth of the error. We need to continue research to find a more effective method for error suppression.

## Figures and Tables

**Table 1:** Main performance parameters of the accelerometers.

Parameter	Index	Unit
Dynamic Range	±15	g
Bias Repeatability	100	μg
Bias Temperature Coefficient	100	μg/°C
Sensitivity Temperature Coefficient	100	ppm/°C
Scale factor	1.2 ± 0.2	mA/g
Temperature Range	−40~+175	°C

**Table 2:** Common calculation methods of well trajectory.

Classification	Methods
Straight line method	Average angle method (AAM)
Broken line method	Balance tangent method (BTM)
Curve method	Corrected average angle method (CAAM)
	Minimum curvature method (MCM)
	Chord step method (CSM)

**Table 3:** MWD prototype Sensor parameters.

Axis	Angular Random Walk (ARW)	Bias Stability (1σ)	
	FOG/(°/√h)	FOG/(°/h)	Accelerometer (m/s^2^)
X	0.0198	0.287	$3.46\times 10^{-4}$
Y	0.0232	0.324	$6.26\times 10^{-4}$
Z	0.0193	0.325	$7.68\times 10^{-4}$

**Table 4:** Surveying methods.

Methods Number	Methods Description
M1	ZUPT
M2	Integrated with drilling pipe length
M3	Proposed algorithm

**Table 5:** Maximum absolute attitude errors.

Methods	Inclination (°)	Toolface (°)	Azimuth (°)
M1	0.0097	0.0802	0.6653
M2	0.0117	0.0470	0.6354
M3	0.0077	0.0230	0.5832

**Table 6:** Maximum absolute position errors.

Methods	East (m)	North (m)	Vertical (m)	Horizontal (m)
M1	53.55	22.05	22.46	57.32
M2	31.60	2.19	4.56	31.67
M3	11.23	1.12	2.34	11.29
